# Autonomic dysfunction after stroke: an overview of recent clinical evidence and perspectives on therapeutic management

**DOI:** 10.1007/s10286-025-01120-0

**Published:** 2025-03-25

**Authors:** Anush Barkhudaryan, Wolfram Doehner, Nadja Jauert

**Affiliations:** 1 Department of Cardiology, Clinic of General and Invasive Cardiology, University Hospital No. 1, Yerevan State Medical University, Yerevan, Armenia; 2 Yerevan Scientific Medical Center, Yerevan, Armenia; 3 Center for Stroke Research Berlin (CSB), Charité-Universitätsmedizin Berlin, Berlin, Germany; 4 German Center for Cardiovascular Research (DZHK), Partner Site Berlin, Berlin, Germany; 5 Berlin Institute of Health-Center for Regenerative Therapies (BCRT), Charité-Universitätsmedizin Berlin, Berlin, Germany; 6 Deutsches Herzzentrum der Charité, Department of Cardiology, Campus Virchow, Charité Universitätsmedizin Berlin, Berlin, Germany; 7 Division of Physiology, Department of Human Medicine, Medical School Berlin (MSB), Berlin, Germany

**Keywords:** Stroke, Autonomic dysfunction, Sympathetic activation, Heart rate variability, Clinical outcome

## Abstract

**Purpose:**

Central autonomic dysfunction is common in acute stroke and is associated with cardiovascular complications and increased mortality. The aim of this review is to present novel diagnostic and therapeutic approaches to the management of this disorder and the latest data on its impact on the clinical outcome after stroke.

**Methods:**

We performed a narrative review of recent literature, with a particular focus on articles related to underlying pathophysiological mechanisms of cardiac autonomic dysregulation, the role of cardiac autonomic dysregulation in the activation of neuroinflammatory response and the development of cardiovascular, respiratory and metabolic complications in patients with ischemic and hemorrhagic stroke.

**Results:**

The assessment of central autonomic dysfunction by non-invasive diagnostic techniques, including heart rate variability and baroreflex sensitivity, has gained wide practical application in recent years, and they may have a predictive role for evaluating disease prognosis. The emerging evidence derived from recent trials demonstrates that the presence of autonomic imbalance may lead to increased mortality and have an adverse effect on post-stroke rehabilitation.

**Conclusion:**

The early detection and treatment of central autonomic system dysfunction may lead to improved survival of patients with stroke. Among the available therapeutic approaches, neuromodulatory techniques and pharmacological interventions are promising strategies which may be implemented as part of standard acute stroke care to improve patient recovery. Future studies are warranted to address the long-term effects of potential therapeutic agents on the modulation of cardiovascular autonomic function in stroke survivors.

## Introduction

Stroke is considered one of the leading global causes of disability worldwide [1], highlighting the ongoing necessity for optimizing treatment strategies leading to improvement of stroke care. The autonomic nervous system (ANS) regulates the functions of body systems and organs, such as the vascular system, arterial blood pressure (BP), cardiac function, fluid control, body weight and homeostasis. This regulation is mediated by the central autonomic network, which consists of cortical and subcortical brain areas and regions of the brainstem acting through sympathetic and parasympathetic nerve fibers, as well as hormones and neurotransmitters [2]. The integrity and function of the ANS may be affected by acute stroke [3], leading to central autonomic dysfunction (AD) in an estimated 25–76% of patients [4–6]. This disorder represents a serious complication which may affect body functions, clinical status and disease outcome.

In this review, we address the pathophysiological mechanisms of post-stroke AD, the systemic manifestations of this disorder, the clinical relevance of contemporary diagnostic methods for the assessment of cardiac autonomic derangement, as well as potential non-pharmacological and pharmacological treatment strategies in this patient cohort. We also discuss the findings of recent clinical studies focused on analyzing the effect of autonomic imbalance on the post-stroke outcome.

## Systemic role of post-stroke central AD

**Fig. 1:**
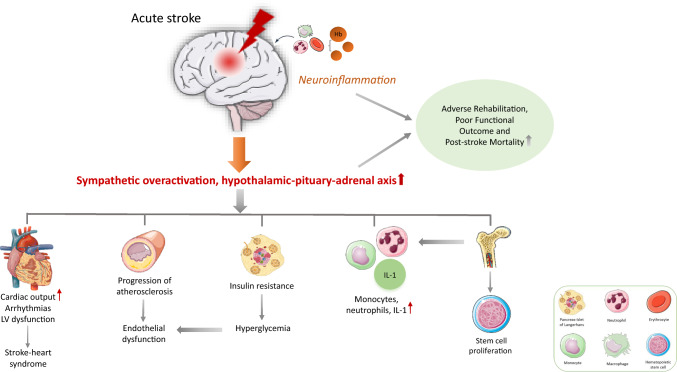
The systemic effects of central autonomic dysfunction in acute stroke. *Hb* hemoglobin, *IL-1* interleukin-1, *LV* left ventricle

Brain injury may lead to the disruption of neuronal connections within the central autonomic network resulting in AD and impaired regulation of BP and heart rate (HR), thereby increasing the risk of post-stroke complications [2]. The impairment of autonomic function is characterized by a decrease in parasympathetic tone and sympathetic activation, leading to the production of catecholamines from the adrenal gland and sympathetic nerve fibers exerting their adverse effects by direct and indirect pathways [7, 8] (Fig. 1). The stimulation of α- and β-receptors by catecholamines leads to an increase in cardiac output, generation of reactive oxygen species (ROS) and coronary vasoconstriction, respectively [9]. The presence of sympathetic overactivity and its effect on disease course have been evaluated in several trials. In particular, one study has shown a persistent increase in epinephrine, norepinephrine and dopamine levels at stroke onset, reflecting sympathetic activation, leading to cardiovascular complications and adverse outcome in this patient cohort [10]. In addition, increased muscle sympathetic nerve activity, as a manifestation of autonomic dysregulation in patients with acute ischemic stroke (AIS), has been associated with immunodepression and post-stroke systemic infections [11].

The direct action of noradrenaline via adrenoreceptors results in apoptosis and subendocardial hemorrhage in the myocardium [12, 13]. One possible consequence is that patients with acute stroke may develop left ventricular (LV) dysfunction due to catecholamine-induced cardiac injury [14]. Moreover, the production of catecholamines triggers the activation of monocytes, neutrophils and the cytokine interleukin 1 (IL-1) from the bone marrow and spleen. This cytokine may induce myocardial inflammation, leading to the synthesis of nerve growth factor by cardiomyocytes and myofibroblasts which in turn causes the growth of sympathetic nerve endings and increases adrenergic effects in patients with stroke [13].

## Pathophysiological mechanisms of post-stroke AD

There is a growing body of evidence on the pathophysiological mechanisms of central AD development in stroke.

The injury to the central autonomic network, including injury to the insular cortex, anterior cingulate cortex and hypothalamus, may disrupt the integration and modulation of autonomic functions, such as cardiovascular regulation and homeostasis. Increased sympathetic dominance, which particularly develops due to damage of the insular cortex, as well as disruption of the neuronal connections in the brain stem within the solitary tract and ventrolateral medulla, is linked to reduced baroreflex sensitivity (BRS), sympathetic/parasympathic imbalance and elevated cardiac risk [15]. Thus, attenuated electrocardiographic RR intervals, as well as high-frequency (HF)- and low-frequency (LF)-powers, indicating loss of cardiac autonomic modulation, have been detected in patients with acute ischemic stroke (AIS) of the middle cerebral artery, ranging from 1 to 21 points on the National Institute of Health Stroke Scale (NIHSS) [16]. In particular, central AD may persist 5 years after stroke, leading to reduced heart rate variability (HRV), impaired cardiac vagal modulation, increased systolic BP variability and higher sympathetic vascular modulation [17].

Furthermore, dysregulation of the hypothalamic-pituary-adrenal (HPA) axis exacerbates autonomic imbalance with increased corticotropin-releasing hormone activity, which contributes to stress-induced autonomic disbalance resulting in altered catecholamine levels and reduced HRV [18]. In particular, higher levels of catecholamine metabolites, adrenocorticotropic hormone and cortisol have been detected in patients with severe ischemic and hemorrhagic stroke (HS) [19, 20]. While the right insular cortex seems to be involved in stress-associated responses mediated by the HPA axis, the left hemisphere is responsible for parasympathetic cardiorespiratory activity. Indeed, the impairment of autonomic control has recently been observed in the right hemisphere infarction to involve the insular cortex accompanied by a more significant increase in norepinephrine levels compared to the left-sided stroke, increasing the risk of acute myocardial infarction and heart failure in this patient cohort [21]. A recent study suggested equal cardiac autonomic response to stimulation of the left and right hemispheres [22].

## Systemic inflammatory activation

The alterations in autonomic control may contribute to systemic inflammatory response in patients with stroke. In particular, the overactivation of sympathetic tone and the HPA axis in the acute phase of the disease may lead to the mobilization of leukocytes from the spleen, as well as increased proliferation and differentiation of hematopoietic stem cells in the bone marrow [23–25] (Fig. 1).

The increased production of immunoactive molecules during the first hours after stroke, induced by cerebral ischemia, triggers a release of pro-inflammatory cytokines, including tumor necrosis factor alpha (TNF-a) and IL-6, by activated immune cells, reflecting the peripheral immune response to stroke [17, 26]. In addition, the infiltration of ischemic lesion with macrophages and neutrophils through the disrupted blood–brain barrier results in a local inflammatory response in the brain [27, 28]. In patients with HS, the lysis of erythrocytes releases hemoglobin and iron, which may induce oxidative stress and neuroinflammation, leading to secondary brain injury and deterioration in cognitive function [29, 30].

The association between inflammation and central AD is known as “neuroimmune cross-talk.” The cholinergic anti-inflammatory pathway plays an important role in the interaction between these two systems. In particular, inflammatory molecules may lead to stimulation of the vagal nerve, which may exert its anti-inflammatory effect by the release of acetylcholine [31]. Inflammation may also influence peripheral neural pathways that act on sympathetic ganglia or feedback loops regulating the function of ANS [32]. In the state of sympathovagal imbalance following acute stroke, increased sympathetic outflow may lead to leukocyte infiltration with myocardial damage, whereas diminished vagal activity may lead to a decreased anti-inflammatory response [32, 33]. This active inflammatory response is succeeded by immunosuppression, associated with a high risk of infections, followed by a prolonged residual inflammation in the chronic phase after stroke [25].

## Cardiometabolic complications of neurovegetative imbalance

The development of AD contributes to the stroke-heart syndrome, which includes acute myocardial injury, acute coronary syndrome, LV dysfunction, arrhythmias and sudden death due to the complex interaction between autonomic cardiac regulatory pathways and inflammatory response [13, 34]. In particular, stroke patients may develop repolarization abnormalities, QT-interval prolongation and increased levels of cardiac troponin T (cTnT) biomarker even in the absence of overt cardiovascular disease [26, 35, 36]. One study showed symmetric and inverted T waves in the electrocardiograms (ECGs) of patients with AIS demonstrating post-stroke myocardial dysfunction [37]. In fact, the occurrence of cardiovascular complications has been reported to be more prevalent in stroke patients with ischemic lesions affecting the right insula of the brain [38, 39].

The development of Takotsubo syndrome, characterized by transient LV dysfunction, has also been observed in 0.5–1.2% and 5–10% of patients with AIS and subarachnoid hemorrhage, respectively [40, 41]. This complication may be caused by an increased sympathetic outflow to the heart due to AD triggered by brain injury. In particular, acute ischemia of the brainstem and insular cortex has been related to the development of this disorder since the insular cortex and medulla are the key centers of cardiac autonomic regulation [42]. The sympathetic overactivity may lead to activation of the renin–angiotensin–aldosterone system, which in turn can exacerbate endothelial dysfunction and cause arterial BP to be elevated. The systemic pro-inflammatory response triggered by damaged neuronal cells can alter sympathetic output of the HPA axis, leading to the overproduction of catecholamines [28]. In addition, the development of myocardial stunning in stroke may be caused by microvascular dysfunction due to impaired regulation of coronary microcirculation mediated by neurons of the brain stem [43]. The occurrence of this syndrome has been associated with a higher rate of in-hospital mortality compared to the general population, necessitating timely management of this disorder in the setting of acute stroke [28].

The autonomic dysregulation may also induce metabolic alterations in patients with acute stroke. The ischemic injury of the brain leading to sympathetic activation may result in hyperglycemia due to elevated levels of circulating catecholamines [13]. The sympathetic activation accompanied by the overproduction of norepinephrine and cortisol may lead to insulin resistance contributing to post-stroke hyperglycemia which has been associated with unfavorable outcome in non-diabetic patients [44, 45] (Fig. 1). The presence of diabetes mellitus in patients with AIS has been shown to increase post-stroke mortality [46]. Furthermore, a reduction in thyroid hormone levels has been observed in these patients due to suppression of the hypothalamic-pituitary-thyroid axis by corticosteroids and a decreased activity of deiodinase enzyme, disrupting the conversion of thyroxin to triiodothyronine [47, 48]. Thus, pre-existing and stroke-related endocrine disorders have been shown to predict adverse functional outcome in stroke survivors [49, 50].

## Peripheral endothelial dysfunction

The presence of central AD may also lead to endothelial dysfunction, particularly in elderly patients, which is characterized by an activation of systemic inflammation [51]. The decrease in an anti-inflammatory effect of the vagal nerve, due to ANS imbalance, increases inflammation and oxidative stress, which may exacerbate endothelial injury [52]. In addition, high levels of circulating catecholamines may contribute to the progression of atherosclerosis and stimulate adrenergic receptors, leading to increased endothelial permeability in these cells [53, 54]. The presence of endothelial dysfunction may be detected by a low reactive hyperemia index as assessed by peripheral arterial tonometry. This vascular damage further increases the risk of recurrent cerebrovascular and cardiovascular adverse events requiring timely therapeutic intervention to prevent progression of this pathology [55].

## Sleep-disordered breathing

The development of sleep-disordered breathing is a common complication detected in more than 70% of patients with acute stroke [56]. This disorder may be caused by damage to the autonomic control of the respiratory function and mainly occurs in the form of obstructive sleep apnea (OSA) [57]. The hypoxemia induced by OSA has been reported to increase sympathetic nervous system activity, leading to the elevation of arterial pressure, hypercoagulability and deterioration of peripheral endothelial function in the patient cohort [58]. In a study in patients with AIS, the authors found an association between sleep-disordered breathing and endothelial dysfunction, demonstrating an interconnection of pathophysiological pathways between central and vascular AD, as well as respiratory complications in this patient cohort [59]. The beneficial effect of positive airway pressure therapy for treatment of this post-stroke complication still needs to be investigated in future trials [60].

Central sleep apnea is the second most common subtype of breathing disorder after stroke, with an incidence of 12%. This form of sleep apnea may develop as a result of airflow cessation due to the absence of respiratory effort [61–63]. Although the prevalence of central apnea is relatively low, particularly in patients with ischemic stroke, its presence has been associated with adverse functional and clinical outcome [52, 64]. A recent clinical study reported that nocturnal hypoxemia and the central sleep apnea index were associated with increased post-stroke mortality in AIS [65].

## Assessment of cardiac AD after stroke

The assessment of AD in patients with acute stroke may be a challenging task for clinicians and is therefore rarely done at bedside. The difficulties encountered in routine diagnostic assessment may be due to the timing of the analysis of autonomic function and various definitions of central AD that render interpretation of the measurement difficult [16]. Evaluation of post-stroke cardiac autonomic function is mainly based on HRV, BP variability (BPV), baroreflex sensitivity (BRS), the Valsalva maneuver and plasma catecholamine levels [66]. Recently, the analyses of HRV and BRS have gained wide clinical application for the detection of impaired autonomic regulation in stroke patients and may be used to predict disease outcome [67, 68].

## Interpretation of HRV

The analysis of HRV is a non-invasive method for assessment of post-stroke cardiac AD that is easy to apply at bedside [69]. The HRV parameters may be obtained from 5-min ECG recordings or 24-h Holter monitoring [70]. The frequency-domain analysis of HRV is based on the identification of LF (range: 0.04–0.15 Hz) and HF oscillations (range: 0.15–0.4 Hz). The power of the HF band is determined by vagal modulation, and that of the LF band is determined by both sympathetic and parasympathetic nervous systems [71, 72]. The LF/HF ratio represents the sympatho-vagal balance [73]. The time-domain analysis includes the standard deviation of NN intervals (SDNN), standard deviation of the 5-min average NN intervals (SDANN), root mean square of successive difference of intervals (RMSSD), percentage of RR intervals > 50 ms apart (pNN50) and the integral of the density of the RR interval histogram divided by its height (HRV triangular index [TI]) [30, 74]. The RMSSD and HF parameters reflect vagal activity, whereas SDNN demonstrates the overall ANS function [52]. The presence of cardiac AD is defined by depressed HRV as revealed by SDNN < 100 ms and HRV TI ≤ 20 [52, 75].

In one study, depressed HRV in 22–57% of patients with stroke was shown [76]. In another study, Yperzeele et al. demonstrated impaired HRV in patients with AIS and HS compared with controls in a systematic review of 22 clinical trials [77]. Most importantly, evaluation of HRV may have prognostic relevance in stroke survivors. In particular, the presence of abnormal HRV in the acute phase of stroke has been associated with adverse outcome during the first 3 months [78]. The analysis of HRV may also allow an evaluation of the risk of post-stroke complications, including neurological status, infarct expansion, in-hospital death and long-term mortality [4, 79, 80]. However, a number of comorbidities and factors may affect the assessment of HRV. In particular, a decrease in HRV may also develop due to physiological factors (such as age, sex and circadian rhythm), lifestyle factors (including smoking, alcohol consumption, increased body mass index [BMI], and regular physical activity), external factors, as well as by concomitant acute and chronic diseases; all of these factors complicate interpretation of this parameter [81]. Therefore, these factors need to be considered during the assessment of HRV, possibly providing an insight into the presence of central AD and allowing administration of treatment in the early stage of stroke.

## Analysis of baroreflex sensitivity

The baroreceptor reflex represents a vasculo-neural feedback loop mechanism for the regulation of arterial BP. The alteration in systemic BP activates baroreceptors in the carotid arteries, right atrium and the aortic arch, which transmit signals by the glossopharyngeal and vagal nerves to the nucleus of the solitary tract (NTS) of the brainstem [10, 82]. The BRS is measured in milliseconds of RR interval duration to each millimeter mercury (mmHg) of arterial BP, with a normal value of about 15 ms/mmHg [83]. The development of AD manifested by sympathetic overactivation leads to post-stroke reduction of BRS, which may develop due to an injury to the insular cortex, an area connected with NTS that regulates autonomic function [84], and contribute to BPV in patients with AIS and acute HS [85, 86]. In particular, a more significant decrease in BRS has been detected in left-sided insular stroke compared to right-sided injury [14]. The authors of one study showed that a decrease in BRS was more expressed in a large hemisphere or brainstem infarction [87]. Evaluation of BRS was performed by considering systolic BP changes > 1 mmHg, sequences longer than 3 beats and a correlation coefficient > 0.85 using a synchronous mode and a shift mode from one to six heart beat shifts. The average slope of the regression lines was selected for the assessment of BRS [75, 88, 89]. Tang et al. have also shown that systolic BPV and impaired BRS, measured from a 5-min beat-to-beat BP and HR monitoring within 7 days from the stroke onset, were associated with adverse outcome [69]. Moreover, patients with reduced BRS had an adverse functional outcome at 1 month and a high incidence of in-hospital complications. In particular, the evaluation of BRS in this study cohort was performed by continuous recording arterial BP and beat-to-beat HR for at least 30 min within 1 week after acute stroke using a validated technique involving synchronization of systolic BP and RR interval data [90].

Previous studies have demonstrated that the presence of vascular calcification, especially in elderly patients, surgical endarterectomy and antidepressant drugs may also lead to BRS reduction, thereby hindering its diagnostic relevance for the detection of AD in this patient cohort [91, 92]. Despite these factors, the non-invasive assessment of HRV and BRS for the detection of cardiac autonomic dysregulation may have a prognostic role in the evaluation of clinical outcome in patients with acute stroke.

## Impact of AD on the clinical outcome after stroke

Central autonomic dysregulation may have an adverse effect on the clinical course of stroke and the efficacy of its treatment [93]. Temporal loss of balanced autonomic control, typically manifested by a decrease in vagal activity and sympathetic dominance in the acute and subacute phases of stroke, has been associated with adverse prognosis [90, 94].

Recent clinical data show that AD may have a prognostic role in the evaluation of post-stroke recovery. The presence of this disorder in the acute phase of stroke may cause BP fluctuations, leading to cerebral hypoperfusion, and worsen neurological rehabilitation [5, 95]. In fact, an association between systolic BP variation and severity of outcome in acute stroke has been demonstrated [96]. Activation of ANS may contribute to immunodepression, thereby increasing the risk of pneumonia and urinary tract infections in about 30% of patients [97–99]. In particular, hypoxia may further deteriorate cerebral perfusion, whereas secondary bacterial infections may activate autoreactive immune responses against central nervous system antigens [99]. However, the role of these factors in the progression of the disease needs to be elucidated in prospective studies.

The effect of vegetative system imbalance on the clinical outcome of patients with acute stroke has been investigated in multiple previous trials [4, 5, 79] (Table 1). In the preliminary study by Nayani et al., the presence of AD was associated with neurological deterioration and cardiovascular complications at 1 year after stroke [4]. In this study, HRV interpretation was performed by Erwing’s battery and autonomic function was evaluated by 24-h Holter ECG analysis [4]. Xiong et al. showed that AD was related to adverse functional outcome 3 months after AIS, underlining the importance of timely detection of this disorder [5]. The presence of cardiac AD has also been shown to cause elevations in arterial BP and increased mortality in acute stroke [79]. Furthermore, the authors of another study showed that decreased SDNN was associated with unfavorable neurological outcome 1 year after ischemic stroke [100].

**Table 1:** The association between heart rate variability parameters and clinical outcome after stroke

Reference	Patient cohort (*n*)	Type of stroke	HRV parameter	Clinical outcome
Nayani et al. [4]	101	AS	SDNN < 100 ms	Cardiovascular complications and adverse outcome at 1 year
Scherbakov et al. [75]	103	IS, HS	SDNN < 100 ms HRV TI ≤ 20	Adverse outcome after 4 weeks of rehabilitation
Tang et al. [78]	227	IS	MSE	Improved outcome in patients with non-AF stroke
Chidambaram et al. [79]	97	AS	LF/HF > 1	Increased morbidity, mortality and hypertension
Zhao et al. [100]	186	IS	SDNN	Adverse neurological outcome after 1 year
Li et al. [121]	5308	IS *or* TIA	SDNN < 88 ms	Neurological dysfunction and stroke recurrence after 90 days

*AF* Atrial fibrillation, *AS* Acute stroke, *HF* high frequency,* HRV* heart rate variability, *HRV TI* heart rate variability triangular index, *HS* hemorrhagic stroke, *LF* low frequency, *IS* ischemic stroke, *MSE* multiscale entropy, *SDNN* standard deviation of NN intervals, *TIA* transient ischemic attack

In summary, the results of investigations conducted to date demonstrate that central autonomic dysregulation has a negative impact on functional outcome and is associated with increased post-stroke mortality. Therefore, the early detection of central AD may provide relevant information to identify patients at risk for cardiovascular complications in this vulnerable population. In this context, assessment of cardiovascular AD may provide guidance on targeted treatment to improve the survival of patients with stroke.

## Therapeutic strategies for the management of post-stroke AD

### Neuromodulatory treatment

At the present time, there is no evidence-based treatment to prevent or reverse central AD as part of the standard therapy for acute stroke or in rehabilitation programs [101]. However, several treatment strategies have been proposed for the management of this complication to improve patient outcome [15]. In particular, neuromodulatory techniques, such as transcranial direct current and magnetic stimulation, as well as slow-paced breathing represent non-pharmacological strategies for the enhancement of motor function in chronic phases of stroke that have been shown to improve BRS and increase HRV [102, 103]. The invasive methods of neuromodulation may also be implemented to improve the efficacy of post-stroke rehabilitation [104]. Stimulation of the vagus nerve (VNS) may exert anti-inflammatory and neuroprotective effects on the brain and also have a modulatory effect on cerebrovascular function [105]. The beneficial effects of non-invasive or invasive VNS on enhancing motor function in stroke patients have been described recently [106]. Moreover, non-invasive VNS may have a potential effect in reducing stroke volume, thereby attenuating neurological deficits and dysphagia, as well as improving upper extremity function in patients with stroke [107, 108]. In particular, the therapeutic effects of VNS may be related to an enhancement of angiogenesis and maintenance of blood–brain integrity [109]. However, complications after invasive VNS, including peritracheal hematoma and vocal cord paralysis, may develop in < 5% of patients, possibly delaying the post-operative recovery of these patients [110]. This method represents a promising therapeutic strategy for the prevention and treatment of central AD related to acute stroke.

## Biofeedback training of HRV

The biofeedback assessment of HRV for the treatment of autonomic dysregulation has recently gained significant interest. This procedure is based on the voluntary control of the higher amplitude of HRV by creating a resonance between cardiac rhythm and respiration [111]. Previous studies have demonstrated the beneficial effect of this procedure for the treatment of AD in patients with chronic diseases [111, 112]. Chang et al. have originally shown that biofeedback training contributed to an increase of HRV, reduced depression and improved cognitive function in patients with AIS [113]. In another trial, the authors reported that implementation of this method was associated with attenuation of neurocardiac autonomic imbalance due to restored parasympathetic activity [101]. Thus, the biofeedback assessment of HRV may be added to the in-hospital treatment regimen of stroke to enhance cardiac autonomic modulation.

### Pharmacotherapy

Pharmacological agents that modulate ANS may be used to prevent the development of arrhythmias, including atrial fibrillation and other types of cardiovascular disorders [114]. Previous trials have shown no beneficial effect of β-blocker therapy on the outcome of patients with acute stroke [115]. However, these studies likely enrolled all stroke patients and did not select patients with AD. Therefore, a promising research strategy might be to explore this therapeutic approach with a better patient selection.

Recent clinical data, however, have shown beneficial effects of this therapy in stroke patients. In one study, the administration of carvedilol, a non-selective β-blocker with additional vasodilative effect, was proposed due to its anti-inflammatory properties [116]. Wang et al. showed that treatment with antihypertensive medications and statins contributed to the recovery of autonomic function at rest 3 months after ischemic stroke [117]. The results of another trial revealed that rapid restoration of autonomic balance was also associated with an early initiation of cardiovascular drugs, particularly angiotensin-converting enzyme (ACE) inhibitors and β-blockers, in patients with AIS [118]. In fact, ACE inhibitors, angiotensin-receptor blockers (ARBs) and statins have been shown to improve the sympathovagal balance and BRS, thereby decreasing the risk of stroke recurrence and enhancing patient survival [119–121]. Furthermore, the effects of cholinergic agonists to improve neurobehavioral deficits, as well as decrease oxidative stress and inflammation have been previously shown in experimental studies [122, 123]. Based on these results, nicotinic acetylcholine receptors have recently been proposed as potential targets for improving the functional recovery in patients with stroke by inducing the modulation of immune cells through activation of the cholinergic anti-inflammatory pathway [124, 125]. Future studies are warranted to evaluate the potential of novel pharmacological agents targeting these receptors on improving autonomic function and neurological rehabilitation in patients with acute stroke.

## Conclusion

The central AD is a common complication in patients with acute stroke associated with adverse functional outcome and increased mortality. The findings of recent studies and meta-analyses underline the potential of early detection and treatment of this disorder to improve post-stroke recovery and survival. Future studies need to investigate the practicability of bedside assessment of ANS dysfunction in acute stroke, the clinical efficacy of non-pharmacological neuromodulatory techniques and pharmacological treatments for the recovery of cardiovascular autonomic regulation in patients with acute cerebrovascular diseases.
