# Correction: Autonomic dysfunction after stroke: an overview of recent clinical evidence and perspectives on therapeutic management

**DOI:** 10.1007/s10286-025-01127-7

**Published:** 2025-06-30

**Authors:** Anush Barkhudaryan, Wolfram Doehner, Nadja Jauert

**Affiliations:** 1 Department of Cardiology, Clinic of General and Invasive Cardiology, University Hospital No. 1, Yerevan State Medical University, Yerevan, Armenia; 2 Yerevan Scientific Medical Center, Yerevan, Armenia; 3 Center for Stroke Research Berlin (CSB), Charité-Universitätsmedizin Berlin, Berlin, Germany; 4 German Center for Cardiovascular Research (DZHK), Partner Site Berlin, Berlin, Germany; 5 Berlin Institute of Health-Center for Regenerative Therapies (BCRT), Charité-Universitätsmedizin Berlin, Berlin, Germany; 6 Deutsches Herzzentrum der Charité, Department of Cardiology, Campus Virchow, Charité Universitätsmedizin Berlin, Berlin, Germany; 7 Division of Physiology, Department of Human Medicine, Medical School Berlin (MSB), Berlin, Germany

**Correction: Clinical Autonomic Research** 10.1007/s10286-025-01120-0

In Table 1 of this article, in first column, the reference numbers were incorrectly published in original publication. The original article has been corrected.

Incorrect version:

**Table 1** The association between HRV parameters and clinical outcome after stroke

**Table:** 

Authors	Patient cohort	Type of stroke	HRV parameter	Clinical outcome
Nayani et al. [2]	101	AS	SDNN < 100 ms	Cardiovascular complications and adverse outcome at 1 year
Scherbakov et al. [58]	103	IS, HS	SDNN < 100 ms HRV TI ≤ 20	Adverse outcome after 4-week rehabilitation
Tang et al. [61]	227	IS	MSE	Improved outcome in patients with non-AF stroke
Chidambaram et al. [62]	97	AS	LF/HF > 1	Increased morbidity, mortality and hypertension
Zhao et al. [76]	186	IS	SDNN	Adverse neurological outcome after 1 year
Li et al. [89]	5308	IS *or* TIA	SDNN < 88 ms	Neurological dysfunction and stroke recurrence after 90 days

*AF* atrial fibrillation, *AS* acute stroke, *BP* blood pressure, *HF* high frequency, *HRV TI* heart rate variability triangular index, *HS* hemorrhagic stroke, *LF* low frequency, *IS* ischemic stroke, *MSE* multiscale entropy, *SDNN* standard deviation of NN intervals, *TIA* transient ischemic attack

Correct version:

**Table 1** The association between HRV parameters and clinical outcome after stroke

**Table:** 

Authors	Patient cohort	Type of stroke	HRV parameter	Clinical outcome
Nayani et al. [4]	101	AS	SDNN < 100 ms	Cardiovascular complications and adverse outcome at 1 year
Scherbakov et al. [76]	103	IS, HS	SDNN < 100 ms HRV TI ≤ 20	Adverse outcome after 4-week rehabilitation
Tang et al. [79]	227	IS	MSE	Improved outcome in patients with non-AF stroke
Chidambaram et al. [80]	97	AS	LF/HF > 1	Increased morbidity, mortality and hypertension
Zhao et al. [101]	186	IS	SDNN	Adverse neurological outcome after 1 year
Li et al. [121]	5308	IS *or* TIA	SDNN < 88 ms	Neurological dysfunction and stroke recurrence after 90 days

*AF* atrial fibrillation, *AS* acute stroke, *BP* blood pressure, *HF* high frequency, *HRV TI* heart rate variability triangular index, *HS* hemorrhagic stroke, *LF* low frequency, *IS* ischemic stroke, *MSE* multiscale entropy, *SDNN* standard deviation of NN intervals, *TIA* transient ischemic attack

